# Effect of Embedment of MWCNTs for Enhancement of Physical and Mechanical Performance of Medium Density Fiberboard

**DOI:** 10.3390/nano11010029

**Published:** 2020-12-24

**Authors:** Waheed Gul, Hussein Alrobei, Syed Riaz Akbar Shah, Afzal Khan, Abid Hussain, Abdullah M. Asiri, Jaehwan Kim

**Affiliations:** 1 Department of Mechanical Engineering, Institute of Space Technology, Islamabad 44000, Pakistan; waheed.gul@mail.ist.edu.pk; 2 Department of Mechanical Engineering, Prince Sattam Bin Abdulaziz University, Alkharj 11942, Saudi Arabia; 3 Department of Mechatronics Engineering, University of Engineering and Technology, Peshawar 25120, Pakistan; rasayed@uetpeshawar.edu.pk; 4 Department of Mechanical Engineering, University of Engineering and Technology, Peshawar 25120, Pakistan; afzalkhan@uetpeshawar.edu.pk (A.K.); abidhussain@uetpeshawar.edu.pk (A.H.); 5 Department of Chemistry, King Abdulaziz University, Jeddah 21589, Saudi Arabia; aasiri2@kau.edu.sa; 6 Center for NanoCellulose Future Composites, Department of Mechanical Engineering, Inha University, Incheon 22212, Korea; jaehwan@inha.ac.kr

**Keywords:** MDF, MWCNTs, UF, thermal conductivity, formaldehyde emission, I.B, MOE, MOR, Ts, WA

## Abstract

In this research work effect of embedment of multiwall carbon nanotubes (MWCNTs) on the physical and mechanical properties of medium density fiberboard (MDF) have been investigated. The MWCNTs were embedded in urea formaldehyde resin (UF) at 0, 1.5%, 3% and 5% concentrations by weight for the manufacturing of nano-MDF. The addition of these nanoparticles enhanced thermal conductivity by 24.2% reduced curing time by 20% and controlled formaldehyde emission by 59.4%. The internal bonding (I.B), modulus elasticity (MOE), modulus of rupture (MOR), thickness swelling (Ts) and water absorption (WA) properties were improved significantly by 21.15%, 30.2%, 28.3%, 44.8% and 29% respectively as compared to controlled MDF.

## 1. Introduction

Medium density fiberboard (MDF) is a natural timber panel manufactured using wood homogeneous fibers or supplementary lingo cellulosic fibers and binders under pressure and temperature [1]. The applications of MDF include furniture industries, flooring, interior domestic construction, tabletops, vanities, speakers, sliding doors, lock blocks, interior signs, displays, table tennis, pool tables, electronic game consoles, kitchen worktops, office work surfaces, educational institutions, laboratories and other industrial products [2]. Wood mix panels offer a uniform advantage to the structure, which can be important for many design applications [3]. Due to poor physical properties, i.e., water absorption and thickness swelling, and mechanical properties, i.e., internal bonding, modulus of elasticity and modulus of rupture, MDF is no longer used in moist and hot environments. Some research has been conducted to improve the physical and mechanical performance of MDF by introducing melamine, wax and other additives [4].

The fibrous nature of wood has made it one of the most appropriate and versatile raw materials for various uses. However, two properties restrict its much wider use, namely dimensional changes when subjected to fluctuating humidity and mechanical strength. Wood may be modified chemically or thermally so that selected properties are enhanced in a more or less permanent fashion. Another option to improve these properties is to exploit the solutions that nanotechnology can offer. The multiwall carbon nanotubes (MWCNTs) of nanotechnology compounds can deeply penetrate into the wood, effectively alter its surface chemistry and result in a high degree of protection against moisture and mechanical strength. In addition, the use of lignocelluloses materials for the production of advanced wood composites is an innovative avenue for research[5].

This research was conducted with the objective to study the influence of MWCNTs on the physical and mechanical performances of MDF. The comparison of nanoparticle concentration with respect to nano-MDF was also investigated. 

In order to investigate the physical and mechanical performance of nano-MDF, a hybrid approach of nanofillers containing alumina, silicon dioxide and zinc oxide in urea formaldehyde (UF) resin glue was carried out by Candan et al. (2015) [6]. Based on the dry weight of natural fibers, the concentration was kept at 0%, 1% and 3%. Physical and mechanical tests were performed and it was concluded that almost bending strength and modulus of elasticity and screw holding properties enhanced significantly. Taghiyari et al. (2016) explored the physical and mechanical properties of MDF via the addition of nano-wollostonite and camel thorn fibers in UF resin in a ratio 90:30. The size of these fibers was in the range of 100 nm−1µm. An extraordinary improvement was observed in the physical and mechanical properties of MDF [7]. Ismita et al. (2017) conducted an experimental study to investigate the mechanical and physical characteristics of MDF in accumulation of UF-Na+ nanofillers at 2.0%, 4.0% and 6.0%. Among the three concentration levels, 6.0% Na+-based resin had improved modulus of rupture, thickness swelling and modulus of elasticity properties by 34.0%, 6.0% and 65.0%, as compared to normal MDF [8]. CaCO_3_ and poly methyl methacrylate nanoparticles were introduced in UF resin by Yipeng Chen et al. (2018). Thermogravemetric analysis along with mechanical properties and thickness swelling were measured. The results showed an incredible improvement in thermal, mechanical and thickness swelling properties [9]. Da Silvaet al. (2019) developed an experimental study to find out the thickness swelling, bacterial and mold resistance properties of MDF with UF-ZnO and melamine formaldehyde and zinc oxide(MF-ZnO) nanofillers. Three samples of each nanofiller-based MDF were tested for physical and biological performance. Among all concentration levels, 0.5% of ZnO with MF-based MDF has the highest values of physical and biological properties [10]. To explore the thermal, physical and mechanical properties of MDF, Alabduljabbar et al. (2020) studied the effect of 0%, 1.5%, 3.0% and 4.5% alumina nanoparticles in the UF resin and explored the effect of Al_2_O_3_ nanoparticles on the physical and mechanical properties of nano-MDF. The resultant internal bonding, modulus of elasticity, modulus of rupture, thickness swelling and water absorption characteristics were boosted up to 16.4%,31%, 22.12%, 40.15% and 37.53% in that order [11].

Although the addition of nanoparticles has been analyzed in various contexts in literature, the aim of this research work is to explore MWCNTs with diverse absorption levels in order to enhance a number of physical and mechanical characteristics, i.e., thermal conductivity, water absorption, thickness swelling, density, formaldehyde emission, internal adhesion, bending strength and modulus of elasticity. The fibrous nature of wood has made it one of the most appropriate and versatile raw materials for various uses. However, two properties restrict its much wider use, namely dimensional changes when subjected to fluctuating humidity and mechanical strength. Wood may be modified chemically or thermally so that selected properties are enhanced in a more or less permanent fashion. Another option to improve these properties is to exploit the solutions that nanotechnology can offer. The MWCNTs of nanotechnology compounds can deeply penetrate into the wood, effectively alter its surface chemistry and result in a high degree of protection against moisture and mechanical strength. In addition, the use of lignocelluloses materials for the production of advanced wood composites is an innovative avenue for research.

## 2. Materials and Methods

### 2.1. Materials

Urea-formaldehyde resin, MWCNTs and natural wood fibers are used as raw materials for manufacturing of nano-MDF. These raw materials are explained in the subgroup below. 

#### 2.1.1. Urea-Formaldehyde (UF) Resin

Urea formaldehyde resin was purchased from Wah chemical company, Pakistan. The physical and chemical characteristics of the UF resin are shown in Table 1.

#### 2.1.2. Multiwall Carbon Nanotubes (MWCNTs)

(MWCNTs) were provided by Guangzhou Hong Material Technology Company Limited, China. The SEM and XRD of MWCNTs can be seen in Figures S1 and S2 in Supplementary Materials section. The motive for the assortment of MWCNTs is their incomparable tensile strength [12] and thermal conductivity [13,14]. The MWCNTs can deeply penetrate into the wood, effectively alter its surface chemistry and result in a high degree of protection against moisture and mechanical strength. In accumulation, they can be chemically improved [15]. These material goods are anticipated to be appreciated in numerous extents of technology, such as microchip technology, optics, composite materials, nanotechnology and further submissions of materials science. The diameter of a MWCNT tube ranges from 20 to 40 nanometers.

#### 2.1.3. Natural Wood Fibers

Poplar wood fibers were received from Ciel Woodworks (Pvt) Ltd., Peshawar, Pakistan. The length of the fibers is in the range of 0.56–1.0 mm.

#### 2.1.4. Fictionalization of MWCNTs

Raw MWCNTs of a definite weight (1.0 g) were added into 50 mL 3:1 mixture (*v*/*v*) of concentrated H_2_SO_4_ (98%) and HNO_3_ (68%) with sonication at 140 °C for 1 h. The attachment of the functional groups on the surface of the MWCNTs was identified using the Fourier transform infrared (FTIR) spectrophotometer Imprestige-21, Shimadzu Corporation, JAPAN (wave number range of 400–4000 cm^−1^), equipped with an attenuated total reflectance (ATR) device (wave number range of 500–4000 cm^−1^ with 20 scanning rate and resolution of 4 cm^−1^) for the confirmation of gelatin-chitosan interaction in the composites.

#### 2.1.5. Raman Spectroscopy 

Raman spectra of the three kinds of MWCNT were recorded on a Renishaw 1000 Raman spectrometer with the wavelength of the Raman laser at 532 nm.

### 2.2. Preparation of UF-MWCNTs Nanofiller

The MWCNTs-UF nanofluid was primed in the materials Science Lab, Institute of Space Technology, Islamabad, Pakistan with the configurations specified in Table 2 dignified in grams.

The nanofluids were blended by weighing 200 g of urea-formaldehyde resin and 0, 1.5, 3 and 5 wt% of MWCNTs of dry weight of fibers. The sonication of the nanofluids was carried out by means of an Ultrasonic Processor UP 400S of Hielscher Ultrasound Technology Company, USA, for 30 min. The samples were signified by MWCNTs_0_, MWCNTs_1_, MWCNTs_2_ and MWCNTs_3_,according to the meditation of MWCNTs.

### 2.3. Nano-MDF Design

The nano-MDF testers were manufactured in panels with sizes 460 × 460 × 15 mm^3^ with densities in the range of 600–750 kg/m^3^. The MWCNTs-UF nanofluids were properly mixed with poplar wood fibers in rotary drum mixtures fibers and a nozzle. A single opening hot press of Burkle, Germany, operated hydraulically, was used for manufacturing of nano-MDF samples. The hot pressing process parameters, i.e., pressure (160 bar) and temperature (175 °C), were kept constant for all testers. The whole press cycle was maintained for4 min. The manufactured samples were treated in a cooling tower for 3 days.

### 2.4. Scanning Electron Microscopy (SEM)

Before being subjected to SEM, a sample of MWCNTs was prepared in the lab and coated with gold by means of a Safematic CCU-010 Gold/Carbon Sputter, UK. SEM was performed through MIRA 3 TESCAN, Czech Republic, at 50,000× for pure urea-formaldehyde resin and MWCNTs-UF with an extreme working voltage of 20 kV. 

### 2.5. Differential Scanning Calorimetry (DSC)

An apparatus Mettler Toledo TGA/DSC-1-star system, USA, was used for differential scanning calorimetry analysis. The temperature range for this device was kept at 0 °C and 400°C with a heat expanding rate of 10 °C/min in a nitrogen stream of 8 mL/min.

### 2.6. Dynamic Mechanical Analysis (*DMA*)

For DMA, the instrument used is the Pyris Diamond DMA (Perkin Elmer, Columbus, USA with the temperature ramp testing method, from 50 °C to 180 °C, at a heating rate of 10 °C·min^−1^. All tests were conditioned with 0.15% deformation and 10 N perpendicular forces, at a frequency of 1.5 Hz and Poisson’s ratio of 0.440. Before performing DMA, the samples were converted into solid states. The samples were kept at 56 × 13 × 3 mm^3^ as per ASTM D4065. The physical state of the sample was cured solid.

### 2.7. Thermal Conductivity of Nano-Composite

The device used in this research work for measuring thermal conductivity is QTM500, Kyoto electronics company, Japan. The temperature range of this instrument is 100 to 1000 °C. The size of the samples was kept at 0.02 × 0.05 × 0.10 m^3^ and the maximum time for measuring thermal conductivity was 2 min, as per ASTM C 1113-99standards [16,17]. The extent range for this instrument was 0.0115–6.15 W/mK. Samples of nanofillers were collected in vials and subjected to the needle of thermal conductivity sensor. Due to the heat transfer mechanism, the thermal conductivity can be measured using a sensor.

### 2.8. Formaldehyde Emission of Nano-Composite

The perforator method followed by the EN-120 standard (1993) was applied for measuring the formaldehyde emission from nano-MDF. A 150 g nano-MDF sample in powder form was put into a glass flask which already contained550 mL toluene. Pure water of about 1000 mL having a pH value of 7 was added into perforator flasks. Toluene was heated up to boiling point, and the vapors generated were then passed from pure water. An ultraviolet spectroscopy was used to examine the formaldehyde emission after it had been treated with acetone.

### 2.9. Statistical Analysis of Nano-Composite

A one-way ANOVA was carried out for statistical investigation origin 8.5, 32-bit software (accessed on 5/10/2020). The Tuckey method was applied with a 95% confidence level to analyze the samples with three iterations of each. The mean value and significant values with variance for each sample were then calculated.

## 3. Results and Discussion

### 3.1. FTIR Analysis of Functionalized MWCNTs

**Figure 1:**
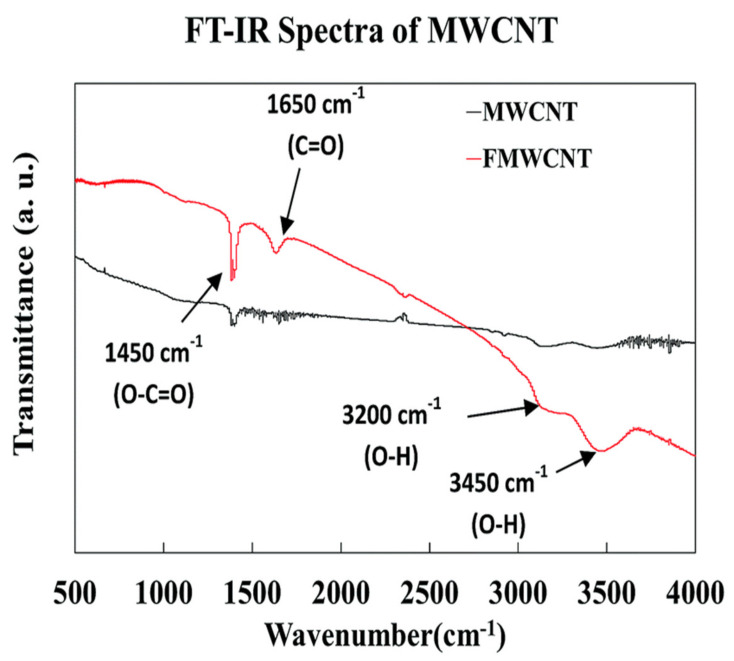
The Infrared (IR) spectra of reference and functional MWCNTs showing hydroxyl group (–OH) and carbonyl group (–CO) attached after acid treatment.

The FTIR spectra of pure and functionalized MWCNTs are shown in Figure 1. From the IR absorption spectra, it was found that the Infrared (IR) absorption spectrum of multi walled carbon nanotubes with function group (MWCNTs-COOH) mainly consisted of hydroxyl group (–OH) stretch at 3200 and 3450 cm^−1^ and carboxyl group (–CO) stretch at 1450 cm^−1^, which are the characteristic peaks and could also be found in the IR spectrum of the raw MWCNTs. However, the intensity of the two peaks in MWCNTs-raw spectrum was much lower than that in the MWCNTs-COOH spectrum. In addition, MWCNTs-COOH has a new peak of small intensity at 1650 cm^−1^, which may be stretching vibrations of carbonyl groups (–CO) as carboxylic groups were formed during the oxidation of hydroxyl compounds [18].

### 3.2. Raman Spectra of Pristine and Modified MWCNTs

**Figure 2:**
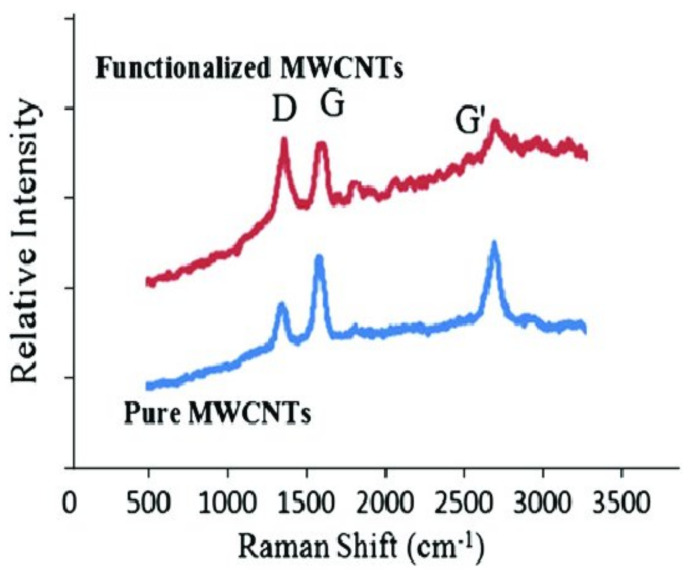
Raman spectroscopy of reference and functional MWCNTs showing –OH and –CO groups attached after acid treatment.

As a very valuable tool to characterize carbon-based nanostructures, the Raman spectra of MWCNT-P and MWCNT-COOH taken at the wavelength of the Raman laser (λ) of 532 nm are shown in Figure 2. For both samples, it presents three main peaks, namely D peak at ~1350 cm^−1^, G peak at ~1590 cm^−1^ and G’ peak at ~2670 cm^−1^. The D peak corresponds to the first-order scattering process of sp^2^ carbons, and it is generally activated by the existence of vacancies, surface functional groups, boundaries and other defects. The G peak derives from the in-plane tangential stretching of –C–C– in graphitic shells, and the G’ peak is the second order of mode of the D peak [19].

### 3.3. Scanning Electron Microscopy of MWCNTs-UF Resin

**Figure 3:**
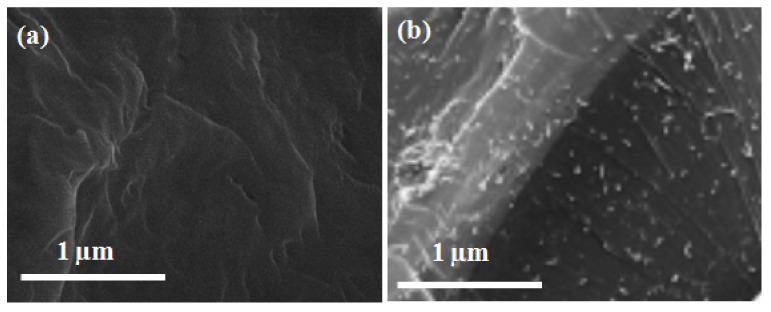
Scanning electron microscopy images of (**a**) pure urea-formaldehyde resin at 50 k and (**b**) MWCNTs-UF resin at 50 k.

In order to investigate the influence of MWCNTs, SEM analysis was carried out for cured UF resin with a MWCNTs concentration of 0% to 5.0%, as shown in Figure 3. An odd structure of linkages of the UF resin was observed, and visible partial pits were examined. These ditches were enclosed by a 5% MWCNTs concentration in UF resin. The strength of the final composite becomes stronger due to the coverage of unwanted cracks and gaps by MWCNTs, as reported by Gul, W.[20]. The visible wires in the scanning electron microscopy demonstrate the attendance of MWCNTs, and the black area represents urea-formaldehyde (UF) resin.

### 3.4. Differential Scanning Calorimetry (*DSC*) of MWCNTs-UF Resin

**Figure 4:**
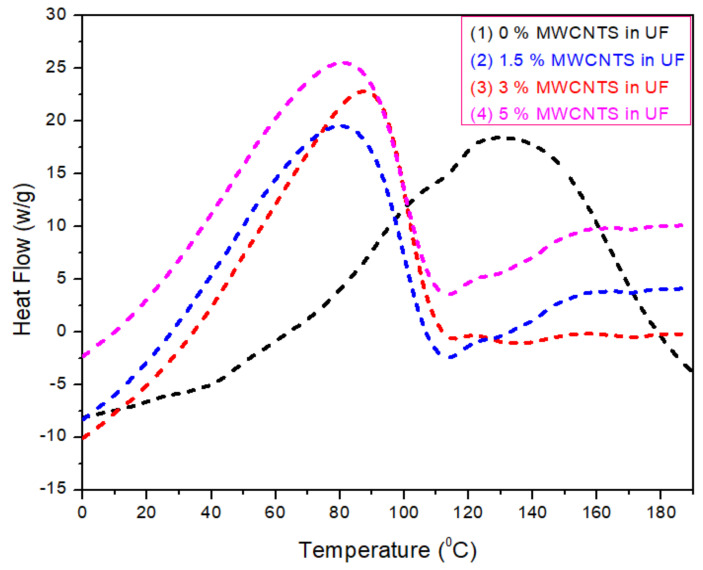
Differential scanning calorimetry of 0%, 5.0%, 1.5%, 3.0% and 5.0% MWCNTs in urea-formaldehyde resin.

The DSC analysis was carried out for 0%, 1.5%, 3% and 5%MWCNTs concentration levels as shown in Figure 4. A demonstration of the relationship between heat flow and temperature is presented for all samples. An inverse relation is observed between curing temperature and MWCNTs concentration. As the concentration of nanoparticles increases, the curing temperature declines, while the amount of total heat content rises linearly with MWCNTs nanoparticles concentration. The peak at 82 °C in 1.5% MWCNTs is obtained due to the additional adhesion formed in UF resin. The same effect had been already shown by other thermosetting resins, as reported by Anuj et al. [21]. From this study, it is also investigated that early curing of the resin occurs due to the addition of MWCNTs. These particles speedup the polymerization process inside the UF resin and ultimately increase the heat transfer rate. A supreme outcome in the form of high production is achieved, which is highly cost effective on a commercial level.

### 3.5. DMA Analysis of UF Resin with and without MWCNTs

**Figure 5:**
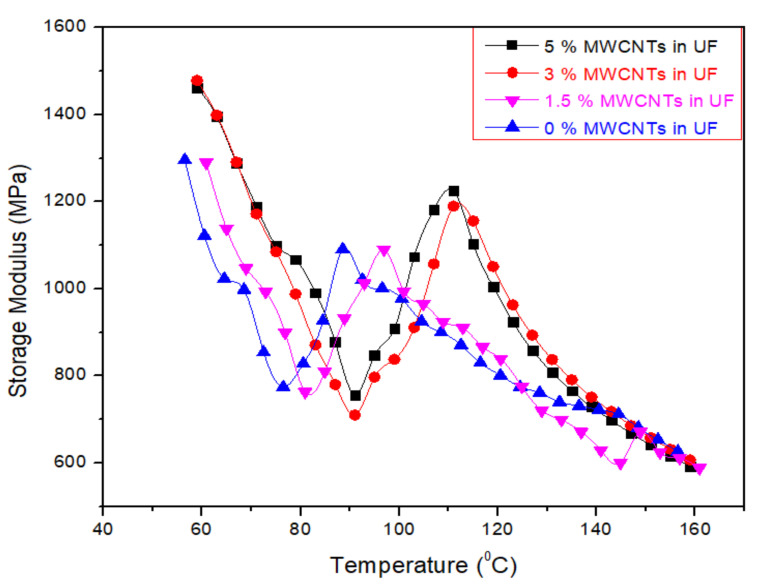
DMA analysis (storage modulus) of UF resin with and without MWCNTs.

The relationship between storage modulus and temperature are described in DMA for the selected four samples, i.e., 0%, 1.5%, 3% and 5% MWCNTs-based UF resin, as shown in Figure 5. The storage modulus values decrease until 140 °C.

**Figure 6:**
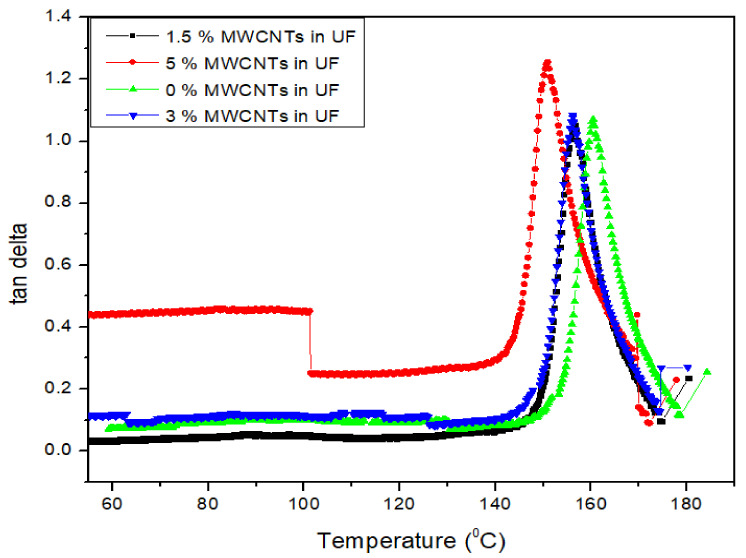
DMA analysis (tan delta) of UF resin with and without MWCNTs.

As the viscosity of the nanofillers increases with the addition of MWCNTs, the gelling occurs early and ultimately increased the storage modulus and tan delta values. The gel time was achieved for 0%, 1.5%, 3.0% and 5% MWCNTs at 87 °C, 87 °C, 96 °C, 110 °C and 112 °C, respectively as shown in Figure 6. When the tan delta decreases, it means that the rate of curing decreases. In a chemical reaction the storage modulus increases up to cross linkages in the resin. The decreasing tan δ can be considered as the rate of curing, as reported by Gupta et al. [22].

### 3.6. Final Physical and Mechanical Characteristics of Nano-MDF

The physical and mechanical characteristics of MDF testers were examined by means of 0%, 1.5%, 3.0%, and 5.0% of MWCNTs and UF glue. Each tester was established for three repetitions and the mean value of individual characteristics as per explication formation was resolute.

These properties are presented in Table 3. The testers were inspected for 0%, 1.5%, 3.0% and 5.0% absorption levels of MWCNTs with three counts of each tester, and the mean values were engaged into deliberation. Equally, thickness Swelling (Ts) and water absorption (WA) investigations were accomplished for 24 h conferring to British Standard EN-3171993 and ASTM D517 correspondingly.

The density rises as the absorption of MWCNTs-UF escalates because of proliferation in the quantity of the nanofluids. A steady decline in the Ts values of the tasters for 24 h was detected, which is owing to a decrease of apertures in nano-MDF panels. Likewise, the WA values correspondingly drop with the upsurge in meditation of MWCNTs-UF resin because of the enhanced drying sheets in the course of hot pressing.

Table 3 summarizes all the physical and mechanical properties of nano-MDF. For 0% MWCNTs in UF resin, the I.B value, which is a reference, reached up to 0.62 MPa. As the meditation level of MWCNTs increases from 0% to 5.0% the I.B value increases from 0.62 to 0.79 MPa in a linear approach and can be compared with EN-319. For 0%, 1.5%,3% and 5% of MWCNTs, the modulus of elasticity comes out to be 2466.6 MPa, 2600 MPa, 3166.67 MPa, 3522.33 MPa, 29.33 MPa, 34 MPa, 36 MPa and 40 MPa, respectively.

The physical properties comprise density, Ts and WA, which were also investigated for 0%, 1.5%, 3%, and 5% MWCNTs concentration levels. The resulting average values of densities were investigated as 697, 718, 721, 724, 720 kg/m^3^ and were compared with the EN-323 standard. The Ts and WA properties were calculated as 22.10%,17.64%,15.63%, 12.19%, 64.21%, 58.15%, 55.34% and 45.57%, respectively. These analyses were accomplished for 24 h according to the EN-317 (1993) standard and ASTM D570 in that order.

A gradual increase for thermal conductivity properties was noted from 0.13% to 0.172%and from 0% to 5% MWCNTs concentration. Finally, the formaldehyde emission for 0%, 1.5%, 3.0% and 5.0%wasrecorded as 15.57, 12.28, 9.27 and 6.32 mg/100 gm.

### 3.7. Statistical Study of Physical and Mechanical Characterstics of Nano-MDF

**Figure 7:**
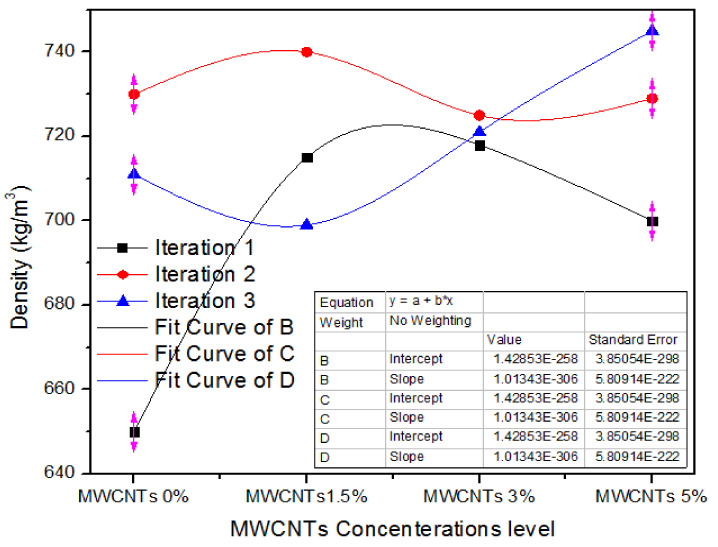
Statistical values of density of MDF for various concentrations of MWCNTs.

Density signifies the mass of medium density fiberboard per unit volume. Figure 7 illustrates the one-way ANOVA consequences of three counts of assessment of density ranging from 0%, 1.5%, 3.0%, and 5% absorption of MWCNTs. For 0% MWCNTs, the three iteration values of density are 650,730 and 711 kg/m^3^. For 1.5% MWCNTs, the three iteration numerical parameters of density are 716,740 and 699 kg/m^3^. In the similar context, for 3% MWCNTs, all the three treatments have 718,725 and 721 kg/m^3^ density parameters. As the absorption level upsurges from 3% to 5%, the density parameters 700, 729 and 745 kg/m^3^ indicate substantial intensification for all iterations.

Table 4 presents the one-way ANOVA statistical method of density parameters for three iterations of MWCNTs (0%), MWCNTs (1.5%), MWCNTs (3.0%), and MWCNTs (5.0%). The 0% MWCNTs comprising medium density fiberboard result in a density average of 697 kg/m^3^ and alteration of 1747, while 1.5%, 3%, and 5% MWCNTs comprising medium density fiberboard have 718, 721.33 and 724 density average values with a variance of 427, 12.33 and 520.33, correspondingly. These density parameters are different from each other and ANOVA significances confirm that *p* is 0.58.

**Figure 8:**
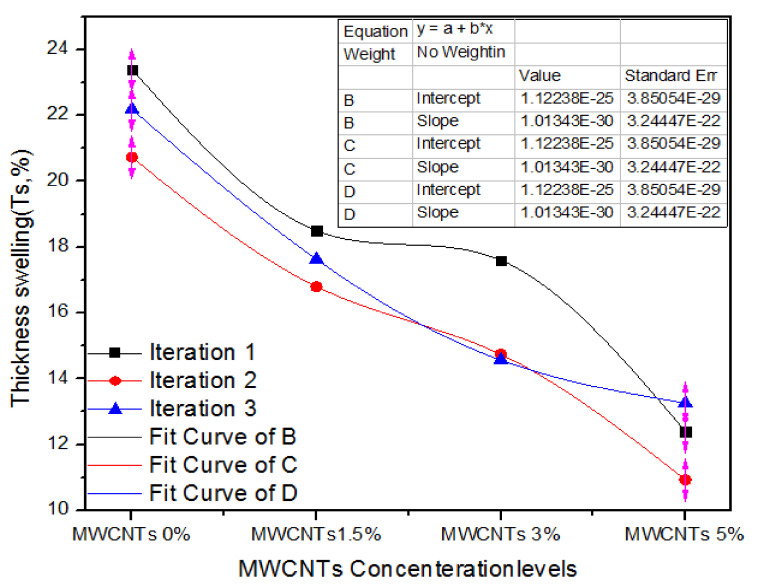
Statistical parameters of thickness swelling of MDF for numerous counts of MWCNTs.

Thickness swelling indicates the solidity characteristics of MDF. Figure 8 expresses the one-way ANOVA outcomes of three iterations of assessment of thickness swelling for 0%, 1.5%, 3% and 5% absorption levels of MWCNTs. For 0% MWCNTs, the three iterations of values of thickness swelling are 22.4%, 20.73% and 22.19% and for 1.5% MWCNTs, and the three iteration values of Ts are 18.5%,17.8%and 16.63%. In the same way, for 3.0% MWCNTs, the three treatments have 17.6%, 14.74% and 14.57% thickness swelling. It might be obvious that as the absorption level upsurges from 3.0% to 5.0%, the thickness swelling values of 12.4%, 10.39% and 13.26% display momentous reduction for all treatments.

Table 5 shows the one-way ANOVA statistical methodology of value of thickness swelling for three iterations of 0%, 1.5%, 3.0% and 5.0% MWCNTs. Firstly, 0% MWCNTs encompassing nano-MDF maintained a thickness swelling average value of 22.10% and variance of 1.78, while 1.5%, 3.0%, and 5.0% MWCNTs_-_based medium-density fiberboard has17.64, 15.63 and 12.19 thickness swelling with average alterations of 0.72, 2.89 and 1.38, respectively. A one-way ANOVA penalty confirms that *p* is equal to 0.000103.

**Figure 9:**
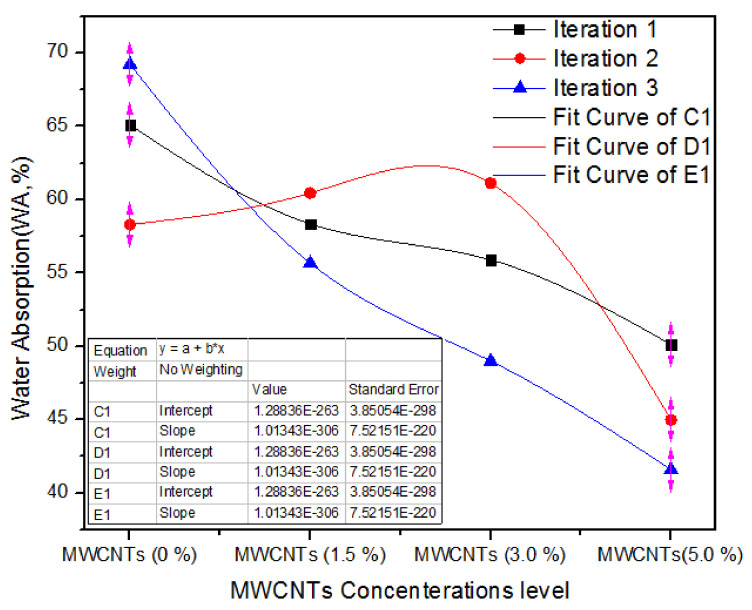
Water absorption of numerous counts of MWCNTs.

Water absorption (WA) is the capability of MDF to absorb water when deep in it. Figure 9 illustrates the one-way ANOVA grades of three iterations of assessment of WA for 0%, 1.5%, 3.0%, and 5.0% absorption levels of MWCNTs. For 0% MWCNTs, the three iterations of parameters of water absorption are 65.1, 58.3 and 69.23%, and for 1.5% MWCNTs, the three iterations of parameters of WA are 58.35, 60.45 and 55.67%. On the contrary, for 3.0% MWCNTs, all the three iterations have 55.9, 61.12 and 49% WA parameters. As the absorption level escalates from 3.0% to 5.0%, the WA values of 50.13, 45 and 41.6% demonstrate a trivial decline for all counts.

Table 6 shows the one-way ANOVA statistical method of WA parameters for three iterations of 0%, 1.5%, 3.0%, and 5.0% MWCNTs. The 0% MWCNTs covering MDF has a WA average value of 64.21% and variance of 30.46, while 1.5%, 3.0%, and 5.0% MWCNTsMDF have 58.15, 55.34 and 45.57% WA average values with variance of 58.15%, 55.34% and 45.57%, respectively. A one-way factor ANOVA ratifies that *p* = 0.0089.

**Figure 10:**
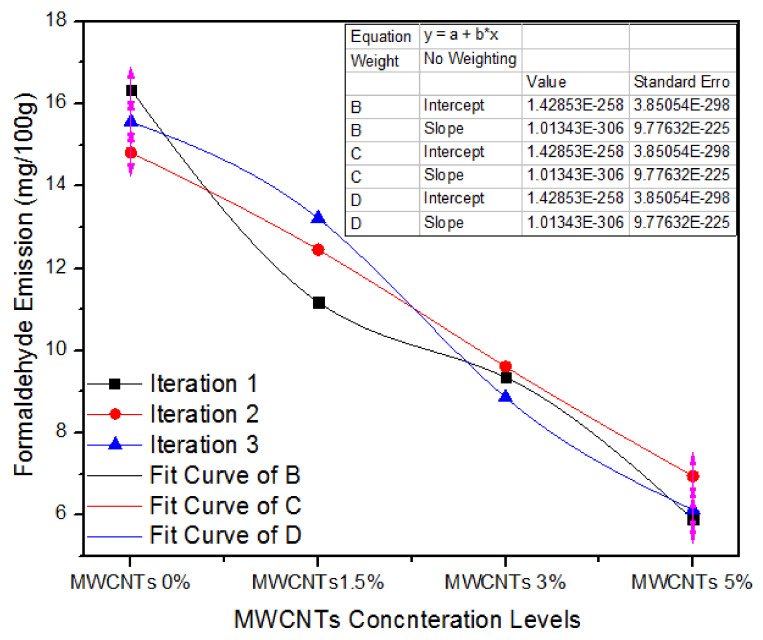
Numerical values of formaldehyde emission of numerous treatments of MWCNTs.

Figure 10 illustrates the one-way ANOVA consequences of three counts of assessment of formaldehyde emission for 0%, 1.5%, 3.0%, and 5% absorption levels of MWCNTs. For 0% MWCNTs, the three iteration values of formaldehyde emission are 16.34, 14.81 and 15.56 mg/100 gm. For 1.5% MWCNTs, the three iterations values of formaldehyde emission are 11.18, 12.45 and 13.21 mg/100 gm. In the similar context, for 3% MWCNTs, all the three treatments have 9.35, 9.61 and 8.86 mg/100 gm formaldehyde emission values. As the absorption level upsurges from 3% to 5%, the formaldehyde emission values of 5.9, 6.95 and 6.13 mg/100 gm indicate substantial intensification for all iterations.

Table 7 shows the one-way ANOVA statistical methodology of formaldehyde emission values for three iterations of 0%, 1.5%, 3.0% and 5.0% MWCNTs. First, 0% MWCNTs encompassing medium density fiberboard have a formaldehyde emission average value of 15.57 mg/100 gm and variance of 0.58, while 1.5%, 3.0%, and 5.0% MWCNTs-based medium-density fiberboard has 12.28, 9.27 and 6.32 formaldehyde emission average values with variances of 1.05, 0.14 and 0.30, separately. These formaldehyde emission values are very close, and the one-way ANOVA penalties authorize *p* = 1.63 × 10^−6^.

**Figure 11:**
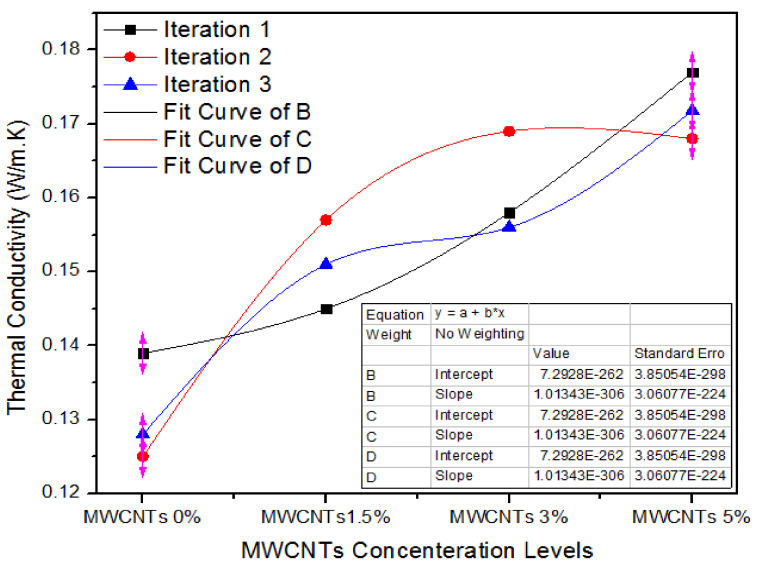
Thermal conductivity of a number of treatments of MWCNTs.

Figure 11 illustrates the one-way ANOVA grades of three iterations of assessment of thermal conductivity values for 0%, 1.5%, 3.0% and 5.0% of MWCNTs.

For 0% MWCNTs, the three iterations values of thermal conductivity are 0.139, 0.125 and 0.128 W/m. K, and for 1.5% MWCNTs, the three iterations values of thermal conductivity are 0.145, 0.157 and 0.151 W/m. K. On the contrary, for 3.0% MWCNTs, all the three iterations have 0.158, 0.169 and 0.156 W/m. K thermal conductivity values. As the absorption level escalates from 3.0% to 5.0% the thermal conductivity values of 0.177, 0.168 and 0.171 W/m. K demonstrate a trivial decline for all counts.

Table 8 shows the one-way ANOVA statistical method of thermal conductivity values for three iterations of 0%, 1.5%, 3.0% and 5.0% MWCNTs.

**Figure 12:**
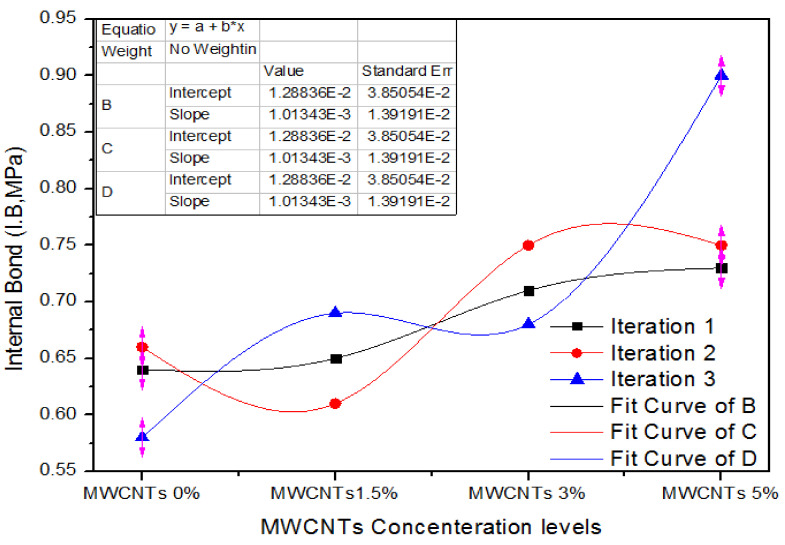
Internal bond values of different counts of MWCNTs.

Figure 12 expresses one-way ANOVA outcomes of addition of MWCNTs in UF resin with 0%, 1.5%, 3.0% and 5% concentrations at three different counts for internal bonding (I.B). These counts are 0.64, 0.66 and 0.58 MPa for 0% MWCNTs. For 1.5% MWCNTs, the measured count parameters are 0.65, 0.61 and 0.69 MPa. Similarly, for 3.0% MWCNTs, the measured counts are 0.71, 0.75 and 0.68 MPa internal adhesion parameters. All the three counts have I.B values of 0.73, 0.75 and 0.9 MPa.

Table 9 shows the statistical analysis for different counts of MWCNTs.

The internal bond values for 0% MWCNTs nano-MDF have an average value of 0.62 MPa and alteration of 0.0017. In contrast, for 1.5%, 3.0% and 5.0% MWCNTs in UF for MDF manufacturing have 0.65, 0.713 and 0.79 average values with variances of 0.0016, 0.0012 and 0.0086, in that order. These internal bonding parameters are corelated with each other and the one-way ANOVA significances approve that the chance (*p*-value) is 0.0296.

**Figure 13:**
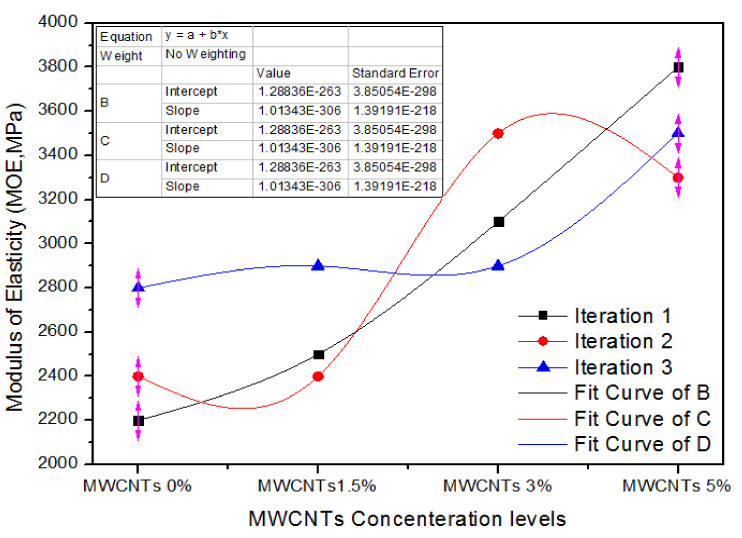
Numerical values of modulus of elasticity (MOE) of various treatments of MWCNTs.

Figure 13 shows the one-way ANOVA consequences of MOE for three counts of evaluation for 0%, 1.5%, 3.0%, and 5.0% concentration levels of MWCNTs. For 0% MWCNTs, the three counts of parameters of MOE are 2200, 2400 and 2800 MPa. For 1.5% MWCNTs, the three treatment values are 2500, 2600 and 2900 MPa. In the same way, for 3.0% MWCNTs, all the three counts have 3100, 3500 and 2900 MPa modulus of elasticity values. The MOE values 3800, 3300 and 3500 MPa express a growth for all counts with 5.0% MWCNTs.

Table 10 comprises the one-way ANOVA analysis of MOE values for three the counts of 0%, 1.5%, 3.0% and 5.0% MWCNTs.

The 0% MWCNTs containing MDF have an average value of 2466.67 MPa and a variance of 93,333.33. In contrast, 1.5%, 3.0%, and 5.0% MWCNTs containing MDF have 2600, 3166.66 and 3533.33 modulus of elasticity mean values with a variance of 70,000, 93,333.33 and 63,333.33, respectively. One-way ANOVA magnitudes endorse that the prospect (*p*-value) is 0.0055.

**Figure 14:**
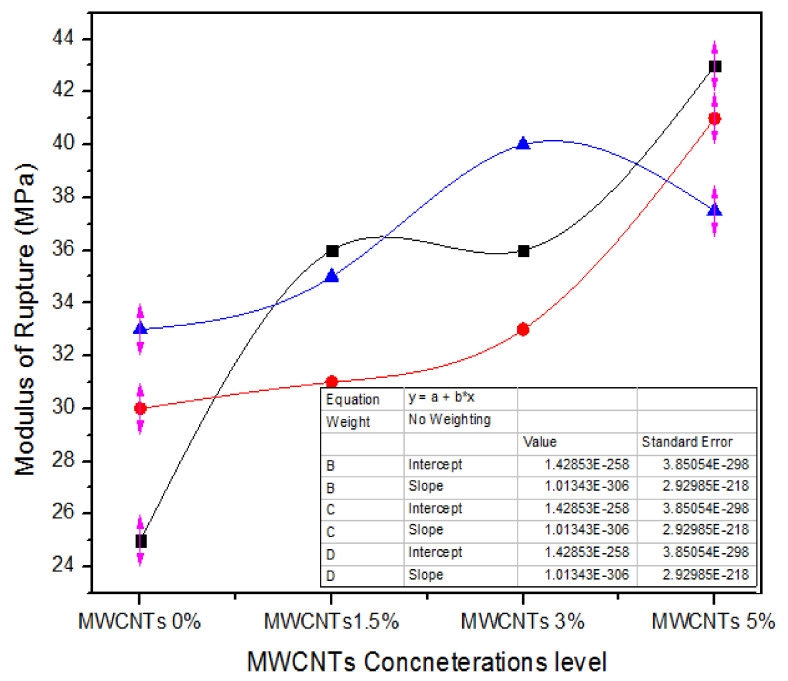
Modulus of rupture vs. % values of counts of MWCNTs.

Figure 14 shows one-way ANOVA results of modulus of rupture (MOR) values of three counts of assessment for 0%, 1.5%, 3.0%, and 5.0% attentiveness levels of MWCNTs. For 0% MWCNTs, the three counts of parameters of MOR are 25, 30 and 33 MPa, while for 1.5% MWCNTs, the three behavior tenets of MOR are 36, 31 and 35 MPa. Similarly, for 3.0% MWCNTs, all the three counts have 36, 33 and 40 MPa MOR parameters. As the absorption concentration rises from 3.0% to 5.0%, the MOR parameters of 43, 41, and 37.5 MPa demonstrate substantial upturn for all iterations.

Table 11 briefly sums up a one-way ANOVA origin-based analysis of MOR parameters for the three counts of 0%, 1.5%, 3.0%, and 5.0% MWCNTs containing MDF. For the 0% MWCNTs concentration, the modulus of rupture mean value was 29.33 MPa and the variance was16.33, while for the 1.5%, 3.0%, and 5.0% MWCNTs concentrations, the modulus of rupture mean values were 3436.33 and 40.5 with alterations of 7, 12.33 and 7.75, respectively. A one-way ANOVA results in *p* = 0.019136.

## 4. Conclusions

Comprehensive research was conducted to find out the effect of MWCNTs added in UF resin for manufacturing of nano-MDF. It has been investigated that the physical and mechanical properties of MDF improved with an extended margin. The Ts and WA properties were enhanced by up to 44.8% and 29% respectively. The addition of these nanoparticles enhanced thermal conductivity by 24.2%, reduced curing time by 20% and controlled formaldehyde emission by 59.4%. Moreover, the internal bond, MOE and MOR properties were improved by 21.15%, 30.2% and 28.3%, respectively. The MWCNTs of nanotechnology compounds can deeply penetrate into the wood, effectively alter its surface chemistry and result in a high degree of improvement in physical and mechanical strength. In addition, the use of lignocelluloses materials for the production of advanced wood composites is an innovative avenue for this research.

A future recommendation is to conduct the research for graphene, alumina and MWCNTs in hybrid mode. This may lead to better results.

## Figures and Tables

**Table 1:** Physical and chemical properties of urea-formaldehyde.

Viscosity (Cps)	Density (kg/m^3^)	pH	Free Formaldehyde (mg/100 g)	Gel Time (s)	Solid Content (%)
180–290	1.25	8.1	0.75	63	59

**Table 2:** Configurations of multiwall carbon nanotubes (MWCNTs)-urea formaldehyde (UF) nanofluid.

	Composition			
Materials	MWCNTs_1_	MWCNTs_2_	MWCNTs_3_	MWCNTs_4_
UF(g)	200	200	200	200
MWCNTs (wt%)	0	1.5	3	5

**Table 3:** The final physical and mechanical properties of 0.016 m MDF samples for diverse absorptions of MWCNTs.

MDF Specimen	Density (kg/m^3^)	TS * (%)	WA * (%)	FE (mg/100 gm)	T.C (W/m.K)	I.B (MPa)	MOE (MPa)	MOR (MPa)
S_0.0_MWCNTs_0.0_	697	22.10	64.21	15.57	0.130	0.62	2466.66	29.33
S_1.5_MWCNTs_1.5_	718	17.64	58.15	12.28	0.151	0.65	2600	34
S_3.0_MWCNTs_3.0_	721.33	15.63	55.34	9.27	0.161	0.71	3166.67	36
S_5.0_MWCNTs_5.0_	724	12.19	45.57	6.32	0.172	0.79	3533.33	40
Standard	720 ± 20	≤12	<45	8.0	≥0.13	0.7 ± 0.03	≥2800	≥25

* 24 h, Moisture Content (Mc) (EN-322) [23] Density (EN-323 standard) [24], Thickness Swelling (TS) (EN-317 standard) [25], Water Absorption (WA) (ASTM D570 standard) [26]. Formaldehyde Emission (FE) EN-120[27]. Internal bonding (I.B) EN-319 [28], Modulus of Rupture (MoR) and Modulus of Elasticity (MoE) EN-310 [29]. Thermal Conductivity (T.C) ASTM C 1113-99 [30].

**Table 4:** Density values of MWCNTs-UF MDF for various iterations.

	Groups	Count	Sum	Average	Variance	
	MWCNTs (0%)	3	2091	697	1747	
	MWCNTs (1.5%)	3	2154	718	427	
	MWCNTs (3.0%)	3	2164	721.33	12.33	
	MWCNTs (5.0%)	3	2174	724.66	520.33	
**ANOVA**						
**Source of Variation**	**SS**	**df**	**MS**	**F**	***p*-Value**	**F Crit**
Between Groups	1398.9	3	466.30	0.68	0.58	4.06
Within Groups	5413.33	8	676.66			
Total	6812.25	11				
Total	6812.25	11				

**Table 5:** Thickness swelling values of MWCNTs-UF MDF for different iterations.

	Groups	Iteration	Sum	Average	Variance	
	MWCNTs (0%)	3	66.32	22.10667	1.787433	
	MWCNTs (1.5%)	3	52.93	17.64333	0.722633	
	MWCNTs (3.0%)	3	46.91	15.63667	2.898233	
	MWCNTs (5.0%)	3	36.59	12.19667	1.388233	
**Source of Variation**	**SS**	**df**	**MS**	**F**	***p*-Value**	**F Crit**
Between Groups	154.13	3	51.37	30.23	0.000103	4.06
Within Groups	13.59	8	1.69			
Total	167.73	11				

**Table 6:** Water absorption values of MWCNTs-UF MDF for various iterations.

	Groups	Iteration	Sum	Average	Variance	
	MWCNTs (0%)	3	192.63	64.21	30.4603	
	MWCNTs (1.5%)	3	174.47	58.15667	5.740133	
	MWCNTs (3.0%)	3	166.02	55.34	36.9588	
	MWCNTs (5.0%)	3	136.73	45.57667	18.43963	
**ANOVA**						
**Source of Variation**	**SS**	**df**	**MS**	**F**	***p*-Value**	**F Crit**
Between Groups	543.0252	3	181.0084	7.904394	0.0089	4.066181
Within Groups	183.1977	8	22.89972			
Total	726.22	11				

**Table 7:** Formaldehyde emission values of MWCNTs-UF MDF for numerous iterations.

	Groups	Count	Sum	Average	Variance	
	MWCNTs (0%)	3	46.71	15.57	0.58	
	MWCNTs (1.5%)	3	36.84	12.28	1.05	
	MWCNTs (3.0%)	3	27.82	9.27	0.14	
	MWCNTs (5.0%)	3	18.98	6.32	0.30	
**ANOVA**						
**Source of Variation**	**SS**	**df**	**MS**	**F**	***p*-Value**	**F Crit**
Between Groups	141.80	3	47.26	90.60	1.63 × 10^−6^	4.06
Within Groups	4.173	8	0.521			
Total	145.98	11				

**Table 8:** Thermal conductivity values of MWCNTs-UF MDF for various iterations.

	Groups	Count	Sum	Average	Variance	
	MWCNTs (0%)	3	0.392	0.130667	5.43 × 10^−5^	
	MWCNTs (1.5%)	3	0.453	0.151	3.6 × 10^−5^	
	MWCNTs (3.0%)	3	0.483	0.161	4.9 × 10^−5^	
	MWCNTs (5.0%)	3	0.5168	0.172267	2.04 × 10^−5^	
**ANOVA**						
**Source of Variation**	**SS**	**df**	**MS**	**F**	***p*-Value**	**F Crit**
Between Groups	0.00282	3	0.00093	23.43	0.000257	4.066
Within Groups	0.00031	8	3.99 × 10^−5^			
Total	0.00312	11				

The 0% MWCNTs covering medium density fiberboard have at thermal conductivity average value of 0.13 W/m. K and variance of 5.43 × 10^−5^, while 1.5%, 3.0%, and 5.0% MWCNTs covering medium density fiber board have 0.15, 0.16 and 0.17 W/m. K thermal conductivity average values with a variance of 3.6 × 10^−5^, 4.9 × 10^−5^ and 2.04 × 10^−5^, respectively. A one-way factor ANOVA ratifies that the prospect (*p*-value) is 0.000257.

**Table 9:** Internal bond values of MWCNTs-UF nano-MDF for a number of iterations.

	Groups	Iteration	Sum	Average	Variance	
	MWCNTs (0%)	3	1.88	0.626667	0.001733	
	MWCNTs (1.5%)	3	1.95	0.65	0.0016	
	MWCNTs (3.0%)	3	2.14	0.713333	0.001233	
	MWCNTs (5.0%)	3	2.38	0.793333	0.008633	
**Source of Variation**	**SS**	**df**	**MS**	**F**	***p*-Value**	**F Crit**
Between Groups	0.050092	3	0.016697	5.059764	0.029688	4.066181
Within Groups	0.0264	8	0.0033			
Total	0.076492	11				

**Table 10:** MOE parameters of MWCNTs-UF nano-MDF for numerous counts.

	Groups	Iteration	Sum	Average	Variance	
	MWCNTs (0%)	3	7400	2466.667	93,333.33	
	MWCNTs (1.5%)	3	7800	2600	70,000	
	MWCNTs (3.0%)	3	9500	3166.667	93,333.33	
	MWCNTs (5.0%)	3	10,600	3533.333	63,333.33	
**Source of Variation**	**SS**	**df**	**MS**	**F**	***p*-Value**	**F Crit**
Between Groups	2,229,166.667	3	743,055.6	9.288194	0.0055	4.066181
Within Groups	640,000	8	0.0033			
Total	2,869,167	11				

**Table 11:** MOR values of MWCNTs-UF MDF for various iterations.

	Groups	Iteration	Sum	Average	Variance	
	MWCNTs (0%)	3	88	29.33333	16.33333	
	MWCNTs (1.5%)	3	102	34	7	
	MWCNTs (3.0%)	3	109	36.33333	12.33333	
	MWCNTs (5.0%)	3	121.5	40.5	7.75	
**Source of Variation**	**SS**	**df**	**MS**	**F**	***p*-Value**	**F Crit**
Between Groups	195.3958	3	65.13194	6.00064	0.019136	4.066181
Within Groups	86.83333	8	10.85417			
Total	282.22	11				

## Data Availability

Data used to support the findings of this study are included within the article.

## Supplementary Materials

The following are available online at https://www.mdpi.com/2079-4991/11/1/29/s1, Figure S1. Scanning electron microscopy images of MWCNTs at. (a) 50,000×, (b) 100,000×; Figure S2. X-ray diffraction analysis of MWCNTs.
