# Supplementary material for: Effect of Embedment of MWCNTs for Enhancement of Physical and Mechanical Performance of Medium Density Fiberboard
Source: Nanomaterials (Basel). 2020 Dec 24;11(1):29. doi: 10.3390/nano11010029 (PMC7824487; doi:10.3390/nano11010029)
Supplement: Supplementary file 1 [file nanomaterials-11-00029-s001.pdf]

nanomaterials

Supplementary Materials

Effect of Embedment of MWCNTs for Enhancement of Physical and Mechanical Performance of Medium Density Fiberboard

Waheed Gul [1], Hussein Alrobei [2,*], Syed Riaz Akbar Shah [3], Afzal Khan [4], Abid Hussain [4], Abdullah M Asiri [5] and Jaehwan Kim [6]

[1] Department of Mechanical Engineering, Institute of Space Technology, Islamabad 44000, Pakistan; waheed.gul@mail.ist.edu.pk
[2] Department of Mechanical Engineering, Prince Sattam Bin Abdulaziz University, Alkharj 11942, Saudi Arabia
[3] Department of Mechatronics Engineering, University of Engineering and Technology, Peshawar 25120, Pakistan; rasayed@uetpeshawar.edu.pk
[4] Department of Mechanical Engineering, University of Engineering and Technology, Peshawar 25120, Pakistan; afzalkhan@uetpeshawar.edu.pk (A.K.); abidhussain@uetpeshawar.edu.pk (A.H.)
[5] Department of Chemistry, King Abdulaziz University, Jeddah 21589, Saudi Arabia; aasiri2@kau.edu.sa
[6] Center for NanoCellulose Future Composites, Department of Mechanical Engineering, Inha University, Incheon 22212, Korea; jaehwan@inha.ac.kr
* *Correspondence:* h.alrobei@psau.edu.sa

Scanning Electron Microscopy of MWCNTs

Scanning electron microscopy (SEM) imaging was used to characterize the surface morphology of multiwalled carbon nanotubes. Figure S1 displays the SEM images of raw MWCNTs. It is evident from the figure that the MWCNTs are cylindrical in shape, curved and tangled together. The dimension of the nanotubes ranges from 20 to 40 nanometers.

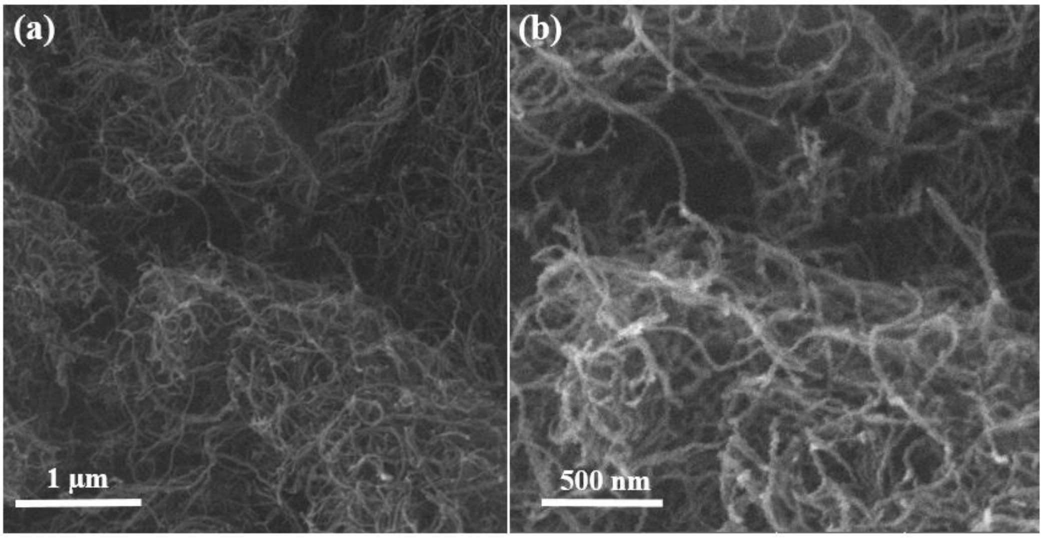

Figure S1. Scanning electron microscopy images of MWCNTs at. (**a**) 50,000X, (**b**) 100,000X.

X-Ray Diffraction Analysis of MWCNTs

Figure S2 shows the X-ray diffraction patterns of raw MWCNTs. From Figure S2 it can be seen that the diffractogram of raw MWCNTs exhibits the typical peaks at 2 h around 26.5 L and 42.7 L, corresponding to the normal structure of graphite (0 0 2) and (1 0 0) reflections (Joint Committee for Powder Diffraction Studies (JCPDS) No. 01-0646) respectively [16]. Similar findings were reported by Chen et al. [17], Gupta et al. [18], Oh et al. [19] and Chen & Oh [20].

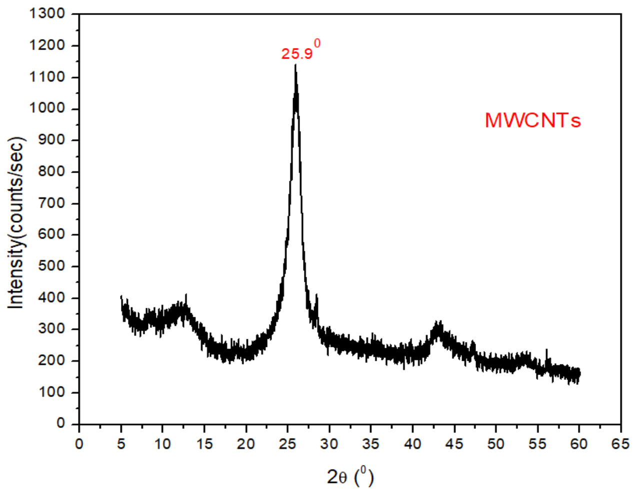

Figure S2. X-ray diffraction analysis of MWCNTs.
